# Alpha-synuclein oligomers and small nerve fiber pathology in skin are potential biomarkers of Parkinson’s disease

**DOI:** 10.1038/s41531-021-00262-y

**Published:** 2021-12-20

**Authors:** Elena Vacchi, Camilla Senese, Giacomo Chiaro, Giulio Disanto, Sandra Pinton, Sara Morandi, Ilaria Bertaina, Giovanni Bianco, Claudio Staedler, Salvatore Galati, Claudio Gobbi, Alain Kaelin-Lang, Giorgia Melli

**Affiliations:** 1 Laboratory for Biomedical Neurosciences, Neurocenter of Southern Switzerland, Ente Ospedaliero Cantonale, Lugano, Switzerland; 2 Faculty of Biomedical Sciences, Università della Svizzera Italiana, Lugano, Switzerland; 3 Neurology Department, Neurocenter of Southern Switzerland, Ente Ospedaliero Cantonale, Lugano, Switzerland; 4 Department of Neurology, Inselspital, Bern University Hospital, University of Bern, Bern, Switzerland

**Keywords:** Parkinson's disease, Diagnostic markers

## Abstract

The proximity ligation assay (PLA) is a specific and sensitive technique for the detection of αSyn oligomers (αSyn-PLA), early and toxic species implicated in the pathogenesis of PD. We aimed to evaluate by skin biopsy the diagnostic and prognostic capacity of αSyn-PLA and small nerve fiber reduction in PD in a longitudinal study. αSyn-PLA was performed in the ankle and cervical skin biopsies of PD (*n* = 30), atypical parkinsonisms (AP, *n* = 23) including multiple system atrophy (MSA, *n* = 12) and tauopathies (AP-Tau, *n* = 11), and healthy controls (HC, *n* = 22). Skin biopsy was also analyzed for phosphorylated αSyn (P-αSyn) and 5G4 (αSyn-5G4), a conformation-specific antibody to aggregated αSyn. Intraepidermal nerve fiber density (IENFD) was assessed as a measure of small fiber neuropathy. αSyn-PLA signal was more expressed in PD and MSA compared to controls and AP-Tau. αSyn-PLA showed the highest diagnostic accuracy (PD vs. HC sensitivity 80%, specificity 77%; PD vs. AP-Tau sensitivity 80%, specificity 82%), however, P-αSyn and 5G4, possible markers of later phases, performed better when considering the ankle site alone. A small fiber neuropathy was detected in PD and MSA. A progression of denervation not of pathological αSyn was detected at follow-up and a lower IENFD at baseline was associated with a greater cognitive and motor decline in PD. A skin biopsy-derived compound marker, resulting from a linear discrimination analysis model of αSyn-PLA, P-αSyn, αSyn-5G4, and IENFD, stratified patients with accuracy (77.8%), including the discrimination between PD and MSA (84.6%). In conclusion, the choice of pathological αSyn marker and anatomical site influences the diagnostic performance of skin biopsy and can help in understanding the temporal dynamics of αSyn spreading in the peripheral nervous system during the disease. Skin denervation, not pathological αSyn is a potential progression marker for PD.

## Introduction

Parkinson’s disease (PD) is a neurodegenerative disease characterized by the presence of pathologic alpha-synuclein (αSyn) deposits in specific areas of the brain. In association with dementia with Lewy bodies (DLB) and multiple system atrophy (MSA), it is part of a spectrum of disorders, the synucleinopathies, characterized by intraneuronal aggregates of αSyn called Lewy bodies and Lewy neurites (PD and DLB) and by glial cytoplasmatic inclusions of αSyn (MSA)^1,2^.

PD is a multisystem disorder that involves both the central and peripheral nervous system^3^. Indeed, while the overt phenotype of PD is that of a movement disorder characterized by bradykinesia in combination with at least one of rest tremor, rigidity, or postural instability, several non-motor autonomic symptoms affect PD patients in prodromal phases^4^. Typical examples are urinary and gastrointestinal dysfunction, impaired sudomotor function, and orthostatic hypotension. Accordingly, in the last decade, multiple studies have shown evidence of αSyn deposits in peripheral nerve tissues and organs, opening the possibility to access pathological tissue in a minimally invasive way^5–7^. This is of utter relevance, considering that the lack of antemortem biomarkers is a major limiting factor for developing specific and effective disease-modifying therapies for neurodegenerative disorders. Furthermore, the clinical heterogeneity of PD and the overlapping clinical spectrum with atypical parkinsonisms (AP), namely MSA and AP with tauopathies (AP-Tau), often prevent a proper diagnosis and management of patients, especially in early phases^8^. In particular, αSyn phosphorylated at Serine 129 (P-αSyn) has been detected in colonic mucosa^9^, submandibular glands^10^, skin^11–14^, and heart^15^ of patients with PD. More recently 5G4 antibody, a conformation-specific monoclonal antibody with high reactivity for aggregated forms of αSyn, and low reactivity for monomeric αSyn^16–18^, has been successfully exploited in the skin^14^, submandibular glands^19^, and colonic mucosa^20^. A systematic meta-analysis on in vivo αSyn detection in peripheral tissues has demonstrated that skin biopsy examination using anti-P-αSyn antibody has the best diagnostic accuracy for PD^21^. Moreover, through intraepidermal nerve fiber density (IENFD) measurement, skin biopsy allowed to demonstrate and quantify small fiber neuropathy in PD^14,22^, which confirms the observation of corneal small nerve fiber reduction in confocal microscopy^23^. However, discrepancies of reported specificity and sensitivity, diverse sites of skin biopsy, and lack of standardized protocols, are major issues in using skin biopsy as a definitive diagnostic tool for PD.

In this study, we exploited proximity ligation assay (PLA), a highly sensitive and specific technique for oligomeric αSyn detection (αSyn-PLA), in skin biopsies of PD in a longitudinal study. PLA is based on proximity probes, composed of oligonucleotide-conjugated antibodies, to recognize a couple of specific targets in close proximity (<16 nm)^24^. αSyn-PLA assay exploits a monoclonal antibody to αSyn with blocking activity on the epitope and preferentially targets αSyn oligomers compared to monomers and fibrils in vitro and binds to early not compacted lesions such us pale bodies, and only rarely to late compacted Lewy bodies in patients’ brain^25^. This is of relevance since αSyn oligomers are early and toxic species implicated in the pathogenesis and spread of disease^26^. The presence of αSyn oligomers was previously detected by αSyn-PLA in the brain of postmortem PD^25^ and MSA^27^, in in vivo gastrointestinal tissues^28^, and very recently, in skin biopsies of PD patients^29^. In particular, Mazzetti et al. described αSyn-PLA within synaptic terminals of cutaneous autonomic fibers in PD and healthy controls (HC), observing a major expression of oligomeric αSyn in patients^29^.

This study aimed to (1) analyze oligomeric αSyn in the ankle and cervical skin biopsies of patients with PD, MSA, AP-Tau, and HC; (2) compare the diagnostic accuracy for PD of αSyn-PLA vs. P-αSyn and vs. 5G4 antibody according to the anatomical site; (3) evaluate pathologic αSyn and IENFD changes by skin biopsy longitudinally at 2 years follow-up in PD.

## Results

### Demographics and clinical data

Demographic characteristics and clinical assessments of the study groups are summarized in Table 1. As expected, AP patients showed a more severe disease than PD, measured by H&Y and MDS-UPDRS, and a higher cognitive impairment, measured by MMSE and MoCA. AP-Tau subjects were significantly older than HC and more depressed than PD. MSA showed a greater autonomic dysfunction measured by COMPASS-31.

**Table 1:** Clinical and skin innervation assessment.

Variable	HC [*n* = 22]	PD [*n* = 30]	MSA [*n* = 12]	AP-Tau [*n* = 11]	Overall *P* value	Pairwise Comparisons					
						PD vs. HC	MSA vs. HC	AP-Tau vs. HC	PD vs. MSA	PD vs. AP-Tau	MSA vs. AP-Tau
Age (years)	60 ± 11	67 ± 11	66 ± 10	73 ± 9	**0.021**	0.072	0.127	**0.001**	1.000	0.131	0.151
Sex (ref. male)	54.4%	60.0%	41.7%	45.5%	0.521	-	-	-	-	-	-
Disease duration (years)	-	4.0 (2.8–8.3)	5.0 (1.3–6.8)	4.0 (3.0–5.0)	0.465	-	-	-	-	-	-
H&Y	-	2.0 (1.0–3.0)	4.0 (3.0–5.0)	4.0 (3.0–4.0)	**<0.000**	-	-	-	**<0.000**	**<0.000**	0.608
MDS-UPDRS-I	-	4.0 (2.0–6.0)	8.0 (7.5–10.5)	12.0 (6.5–18.5)	**0.006**	-	-	-	**0.009**	**0.016**	0.310
MDS-UPDRS-II	-	5.0 (2.5–9.8)	15.0 (9.5–21.0)	24.0 (13.5–34.0)	**0.002**	-	-	-	**0.022**	**0.001**	0.322
MDS-UPDRS-III	-	17.0 (11.3–22.3)	30.0 (23.0–46.5)	39.0 (25.5–59.0)	**<0.000**	-	-	-	**0.001**	**<0.000**	0.573
MDS-UPDRS-Total	-	26.5 (15.3–36.0)	53.0 (36.5–67.0)	90.0 (44.0–112.5)	**0.001**	-	-	-	**0.013**	**0.001**	0.310
COMPASS-31 OH	-	8.0 (0.0–16.0)	24.0 (16.0–30.0)	8.0 (0.0–28.0)	**0.030**	-	-	-	**0.007**	0.674	0.131
COMPASS-31 VM	-	0.0 (0.0–0.0)	0.0 (0.0–0.0)	0.0 (0.0–0.0)	0.353	-	-	-	-	-	-
COMPASS-31 SM	-	0.0 (0.0–4.2)	2.1 (0.0–2.1)	0.0 (0.0–2.1)	0.519	-	-	-	-	-	-
COMPASS-31 GI	-	0.9 (0.0–6.5)	5.4 (3.6–6.7)	0.0 (0.0–6.3)	0.065	-	-	-	-	-	-
COMPASS-31 BL	-	0.0 (0.0–0.0)	1.1 (1.1–2.2)	0.0 (0.0–0.0)	**0.000**	-	-	-	**0.000**	0.805	**0.000**
COMPASS-31 PM	-	0.0 (0.0–0.0)	0.0 (0.0–0.0)	0.0 (0.0–0.0)	1.000	-	-	-	-	-	-
COMPASS-31 Total	-	14.2 (0.0–24.8)	32.3 (22.2–38.2)	12.0 (4.2–28.0)	**0.026**	-	-	-	**0.010**	0.942	**0.020**
BDI-II	-	6.0 (3.0–8.5)	6.0 (3.0–14.0)	14.5 (10.8–17.0)	**0.002**		-	-	0.423	**<0.000**	**0.029**
MMSE	-	30.0 (29.0–30.0)	29.0 (25.0–30.0)	28.0 (23.0–28.0)	**<0.000**	-	-	-	**0.029**	**<0.000**	0.131
MoCA	-	27.0 (24.0–29.0)	25.0 (19.5–26.7)	20.0 (16.0–24.0)	**0.004**	-	-	-	0.095	**0.001**	0.109
Olfactory test	-	6.0 (4.0–8.3)	9.0 (7.5–10.3)	7.0 (4.5–8.5)	0.073	-	-	-	-	-	-
RBD questionnaire	-	3.0 (1.0–5.0)	4.0 (1.0–5.8)	2.0 (0.0–4.0)	0.547	-	-	-	-	-	-
LEDD (mg)	-	495.0 (202.5–737.5)	312.0 (143.0–468.8)	250.0 (125.0–452.0)	0.486	-	-	-	-	-	-
IENFD total (Fibers/mm)	14.5 (10.8–17.1)	11.2 (9.5–14.7)	11.6 (10.0– 12.7)	15.2 (9.2–18.7)	**0.029**	**0.017**	**0.013**	0.947	0.522	0.103	0.079
IENFD cervical (Fibers/mm)	20.8 (15.6–26.4)	16.7 (12.3–23.1)	18.1 (13.5–21.3)	21.5 (15.2–24.7)	0.194	**-**	**-**	**-**	**-**	**-**	**-**
IENFD ankle (Fibers/mm)	8.6 (6.8–10.9)	5.4 (4.3–9.7)	5.9 (3.7–7.3)	7.5 (4.8–10.7)	**0.033**	**0.017**	**0.005**	0.298	0.589	0.417	0.387

*HC* healthy controls, *PD* Parkinson’s disease, *MSA* multiple system atrophy, *AP-Tau* atypical parkinsonisms with tauopathies, *H&Y* Hoehn and Yahr scale, *MDS-UPDRS* movement disorder society-inified Parkinson’s disease rating scale I, II, III and total, *COMPASS-31* composite autonomic symptom scale 31, *OH* orthostatic hypotension, *VM* vasomotor, *SM* sudomotor, *GI* gastrointestinal, *BL* bladder, *PM* pupillomotor, *BDI-II* Beck depression inventory-II, *MMSE* mini-mental state examination, *MoCA* Montreal cognitive assessment, *RBD* Rem behavior disorder, *LEDD* levodopa equivalent dose, *IENFD* intraepidermal nerve fiber density.

*P* values < 0.05 were considered significant and shown in bold.

**Fig. 1:**
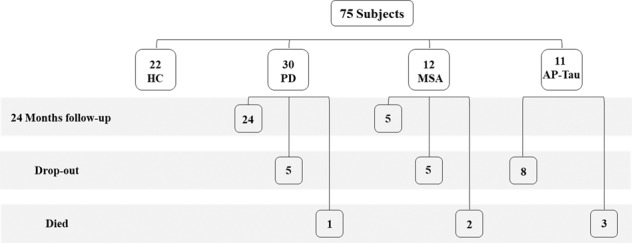
Flowchart of the study population. Study groups are shown longitudinally in time.

After 24 months, 6 patients died (one PD, two MSA, and three AP-Tau), 18 patients dropped out of the study (five PD, five MSA, and eight AP-Tau). Twenty-four of the 30 PD patients underwent a 24-month follow-up analysis (T24) (Fig. 1). The six PD patients who did not perform the T24 assessment were significantly more affected than the rest of the PD group (H&Y, *P* = 0.050; MDS-UPDRS-III, *P* = 0.031) (Supp. Table 1). Since few subjects with MSA and none with AP-Tau performed the T24 evaluation, we did not perform statistical analysis at T24 in these groups.

### PD and MSA display a small nerve fiber pathology

PD and MSA showed a significant reduction of total and ankle IENFD compared to HC (Table 1). A tendency towards a reduced IENFD also at the cervical site was present in PD, even if not significant, possibly due to a higher variability.

### PD and MSA show high positivity for αSyn-PLA

**Fig. 2:**
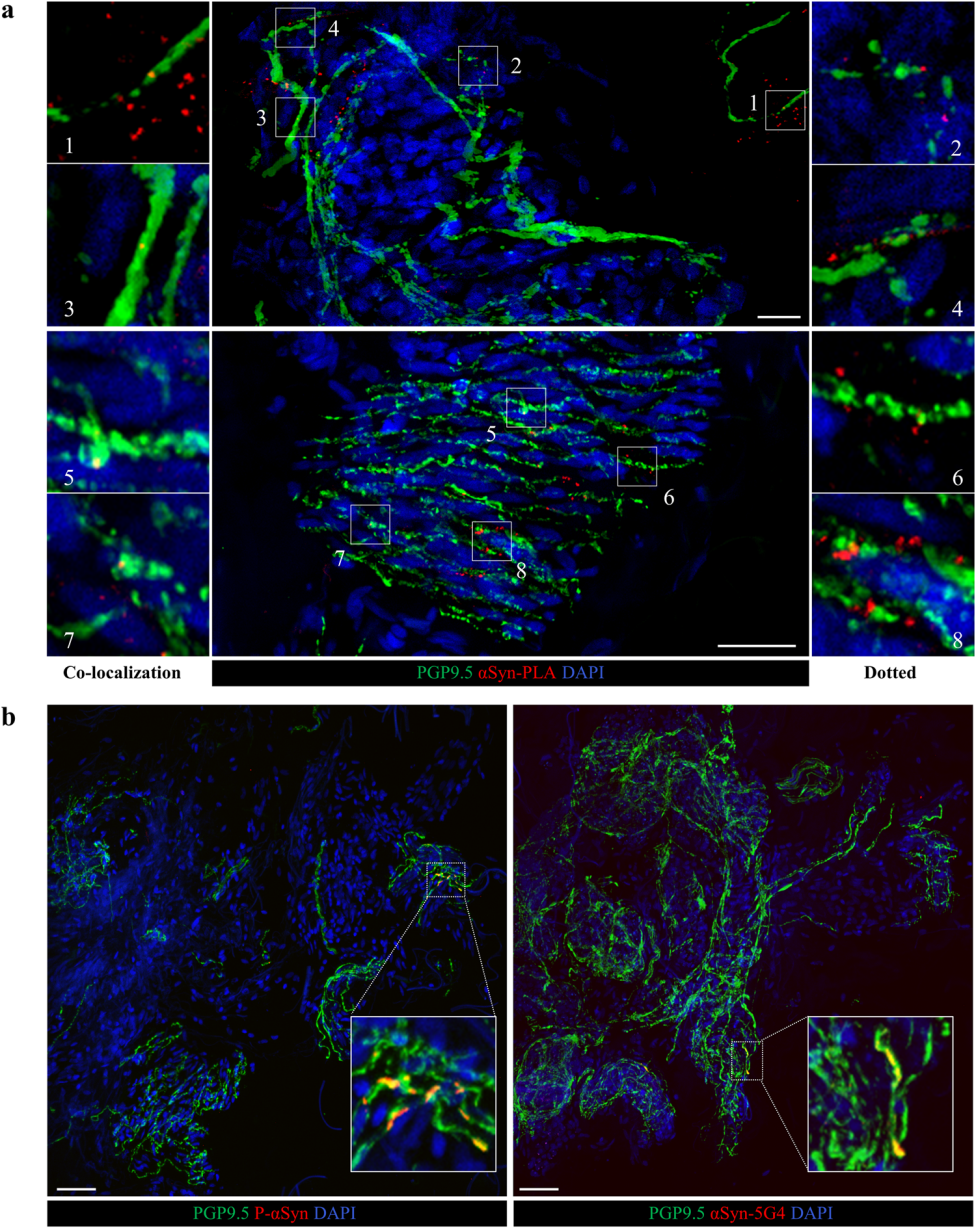
αSyn PLA, P-αSyn and αSyn-5G4 signals in skin. **a** Confocal images of a sweat gland (top) and muscle arrector pili (bottom) in skin biopsy at the cervical site of a PD patient: in green the panaxonal marker protein gene product 9.5 (PGP9.5), in red αSyn oligomers detected by proximity ligation assay (PLA) technique. The Co-localization signal of αSyn-PLA and nerve fibers (magnification on the left) was defined by a yellow signal. The dotted signal (magnification on the right) was defined by an αSyn-PLA signal in the proximity of degenerating nerve fibers, characterized by swelling and fragmentations, or interposed between nerve fiber breakpoints. Scale bar 50 µm. **b** Confocal images of sweat glands in cervical skin biopsy of a PD patient. In red, on the left phosphorylated αSyn, and on the right aggregated αSyn detected by 5G4 antibodies. Co-localization of P-αSyn/PGP9.5 and αSyn-5G4/PGP9.5 is shown in yellow. Scale bar 50 µm.

**Fig. 3:**
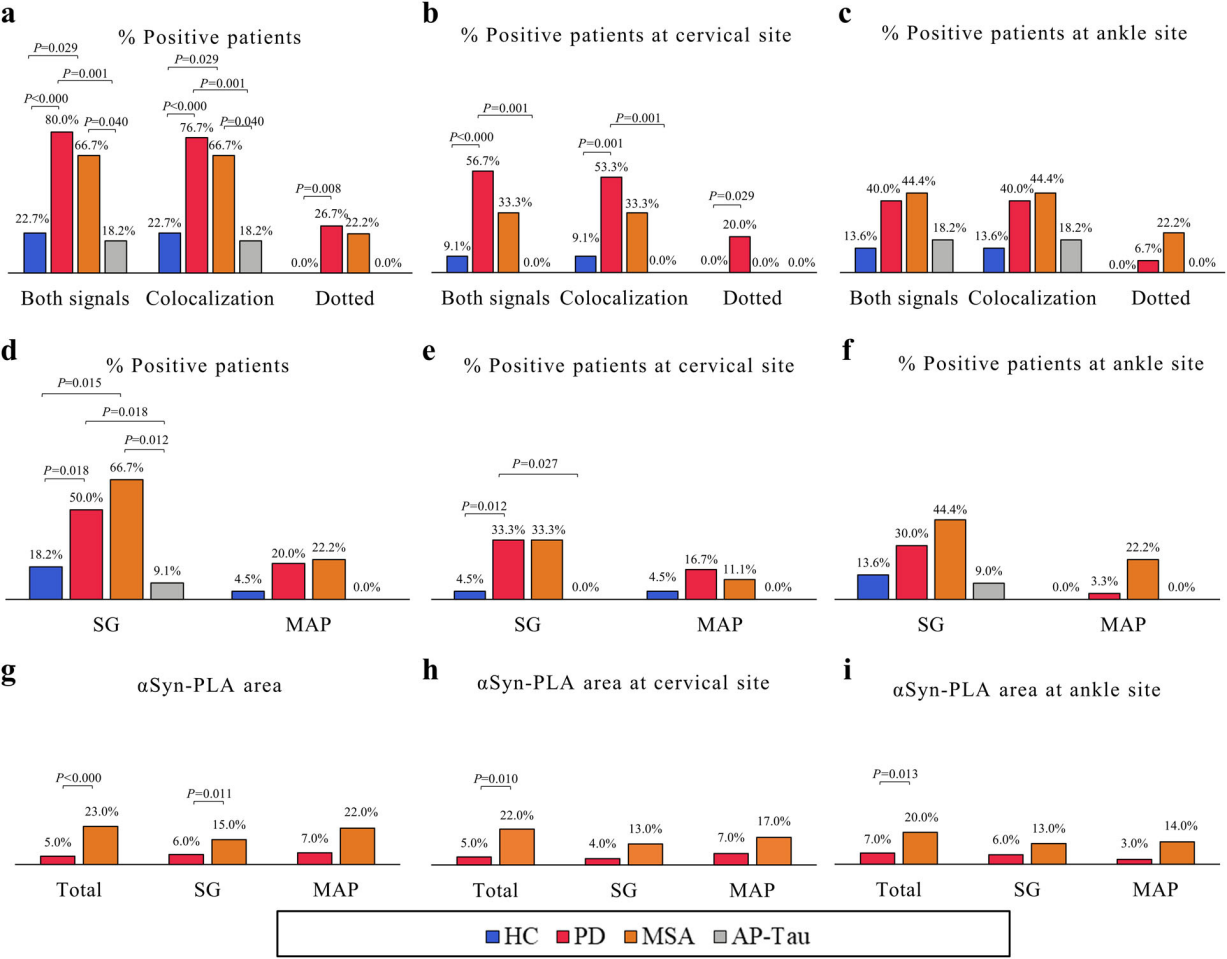
αSyn-PLA signal is significantly more expressed in PD and MSA. **a**–**c** αSyn-PLA signal is higher in PD and MSA. **d**–**f** The signal is observed mainly in the autonomic fibers surrounding SG and to a lesser extent MAP independently from the anatomical site. **g**–**i** The area of intra-nervous oligomeric αSyn is significantly higher in MSA patients than PD, at both sites.

The co-localization signal, defined as an αSyn-PLA signal within the PGP9.5 positive nerves (Fig. 2a), was the most frequent, while the dotted one, defined as the signal located in proximity to degenerated nerve fibers or interposed between nerve fiber breakpoints (Fig. 2a), was observed exclusively in PD and MSA, not in AP-Tau or HC (Fig. 3a). In the MSA group, the dotted signal was always observed in association with the co-localization signal, while in the PD group 3, 3% of patients showed a dotted signal only (Sup. Table 2).

The percentage of patients showing αSyn-PLA was significantly higher in PD and MSA groups compared to HC and AP-Tau (Fig. 3a–c and Sup. Table 2). Considering the two anatomical sites separately, the cervical area allowed the differentiation between PD and HC and AP-Tau (Fig. 3b). No differences were observed between groups at the ankle site (Fig. 3c). MSA showed a higher positivity at the ankle than at the cervical area (Fig. 3c). A double positivity at both anatomical sites was observed only in PD and MSA groups (16.7 and 11.1% respectively).

The majority of αSyn-PLA was detected in nerve fibers surrounding autonomic structures. In 2 out of 30 PD patients, the signal was observed in dermal nerve bundles. Most of the signal was evident in SG innervation (Fig. 3d and Sup. Table 2): PD and MSA group displayed a greater positivity compared to HC and AP-Tau, considering both localization and the cervical site alone (Fig. 3e). No differences between groups in PLA signal were observed in MAP.

MSA displayed a greater area of αSyn-PLA signal within nerves compared to PD (Fig. 3g–i and Sup. Table 3), especially at the cervical site. No differences were observed in the number of αSyn-PLA signals (Sup. Table 3).

Finally, the presence of αSyn-PLA was associated with the diagnosis of PD and MSA by univariate logistic regression analysis (Table 2). PD showed higher odds ratios compared to MSA, except for αSyn-PLA in SG. Moreover, an association with PD diagnosis was observed considering αSyn-PLA signal at cervical site only.

**Table 2:** Association between αSyn-PLA detection and clinical diagnosis.

PD vs. HC		*P* value	Odd ratio	95% CI
Both localizations	All structures	**0.000**	13.60	3.56–51.92
	SG	**0.023**	4.500	1.23–16.49
Cervical	All structures	**0.002**	13.08	2.58–66.28
	SG	**0.032**	10.5	1.23–89.68
PD vs. AP-Tau		*P* value	Odd ratio	95% CI
Both localizations	All structures	**0.001**	18.00	3.05–106.12
	SG	**0.038**	10.000	1.134–88.167
MSA vs. HC		*P* value	Odd ratio	95% CI
Both localizations	All structures	**0.028**	6.800	1.223–37.497
	SG	**0.014**	9.000	1.550–52.266
MSA vs. AP-Tau		*P* value	Odd ratio	95% CI
Both localizations	All structures	**0.037**	9.000	1.140–71.038
	SG	**0.018**	20.000	1.676–238.630

*P* values < 0.05 are considered significant and shown in bold.

### αSyn-PLA showed higher diagnostic accuracy for PD and MSA than P-αSyn and αSyn-5G4

**Fig. 4:**
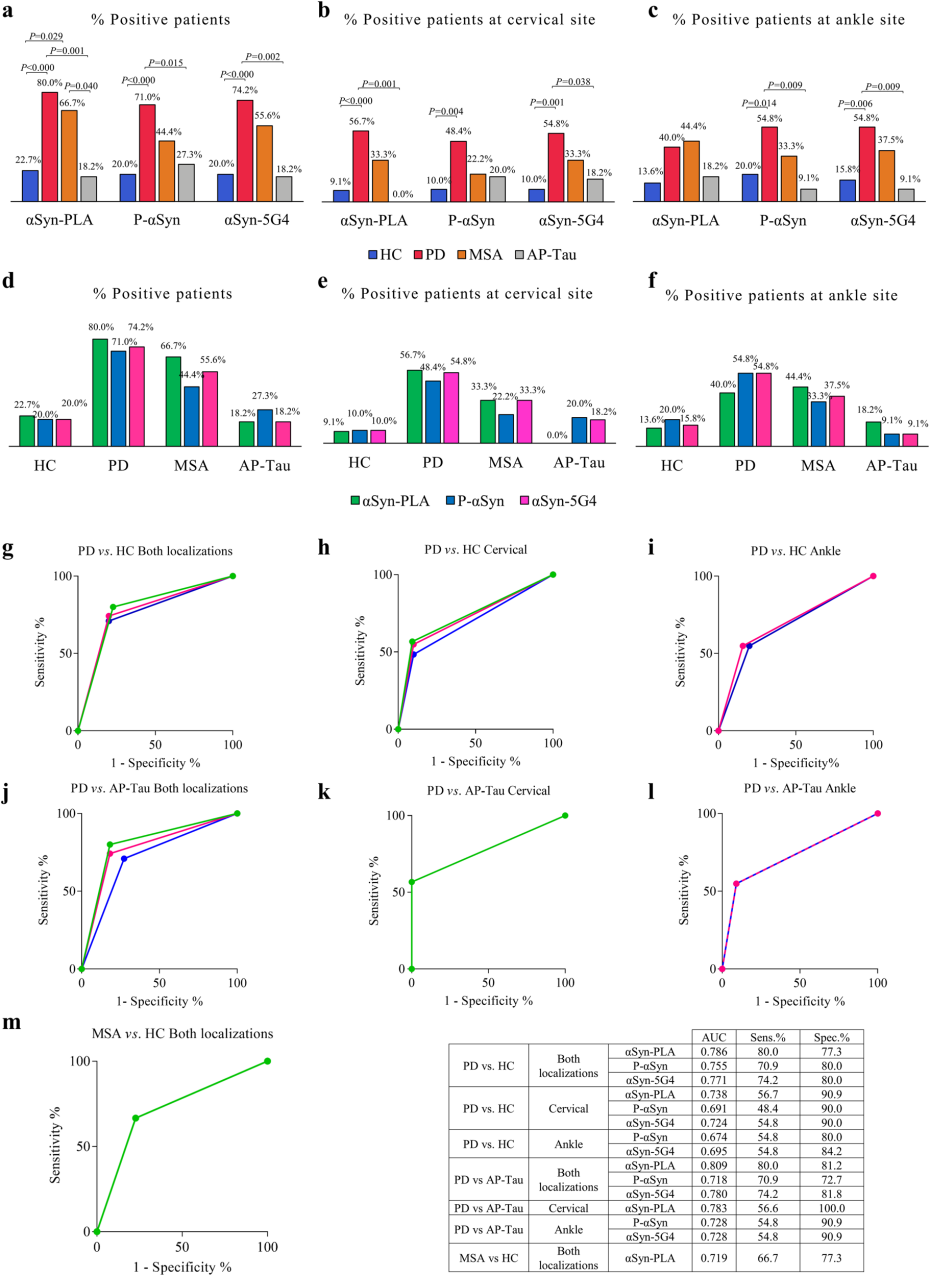
Comparative analysis and diagnostic performance of αSyn-PLA, P-αSyn, and 5G4. **a** αSyn-PLA presented the highest sensitivity for PD and MSA at both locations and allowed the differentiation from HC and AP-Tau. **b** At cervical site αSyn-PLA and αSyn-5G4 showed higher sensitivity than P-αSyn for PD. **c** In the ankle, P-αSyn and αSyn-5G4 were more sensitive for PD then αSyn -PLA. **d**–**f** Within groups no differences were observed between markers, but αSyn-PLA showed a higher sensitivity for MSA than P-αSyn and αSyn-5G4. **g**–**i** ROC curve analysis of PD vs. HC, **j**–**l** PD vs. AP-Tau, **m** MSA vs. HC. For each marker and location, AUC, sensitivity, and specificity are reported.

In addition to αSyn-PLA, subjects were investigated for P-αSyn and αSyn-5G4. All markers were more expressed in PD than HC and AP-Tau, but αSyn-PLA displayed higher sensitivity when considering both locations and the cervical area (Fig. 4a–c and Sup. Table 6). Within groups, no differences were displayed between the three markers (Fig. 4d–f). The ROC curve analysis demonstrated that αSyn-PLA had the highest AUC when considering both locations or the cervical site (4g, h, j, k). Nevertheless, at the ankle, only P-αSyn and αSyn-5G4 could discriminate between groups (Fig. 4i–l), even if with a lower AUC than αSyn-PLA in the cervical area. Finally, only αSyn-PLA allowed the discrimination between MSA and HC, considering both anatomical sites together (Fig. 4m).

### PD patients present a progression of denervation not of pathological αSyn at T24

**Fig. 5:**
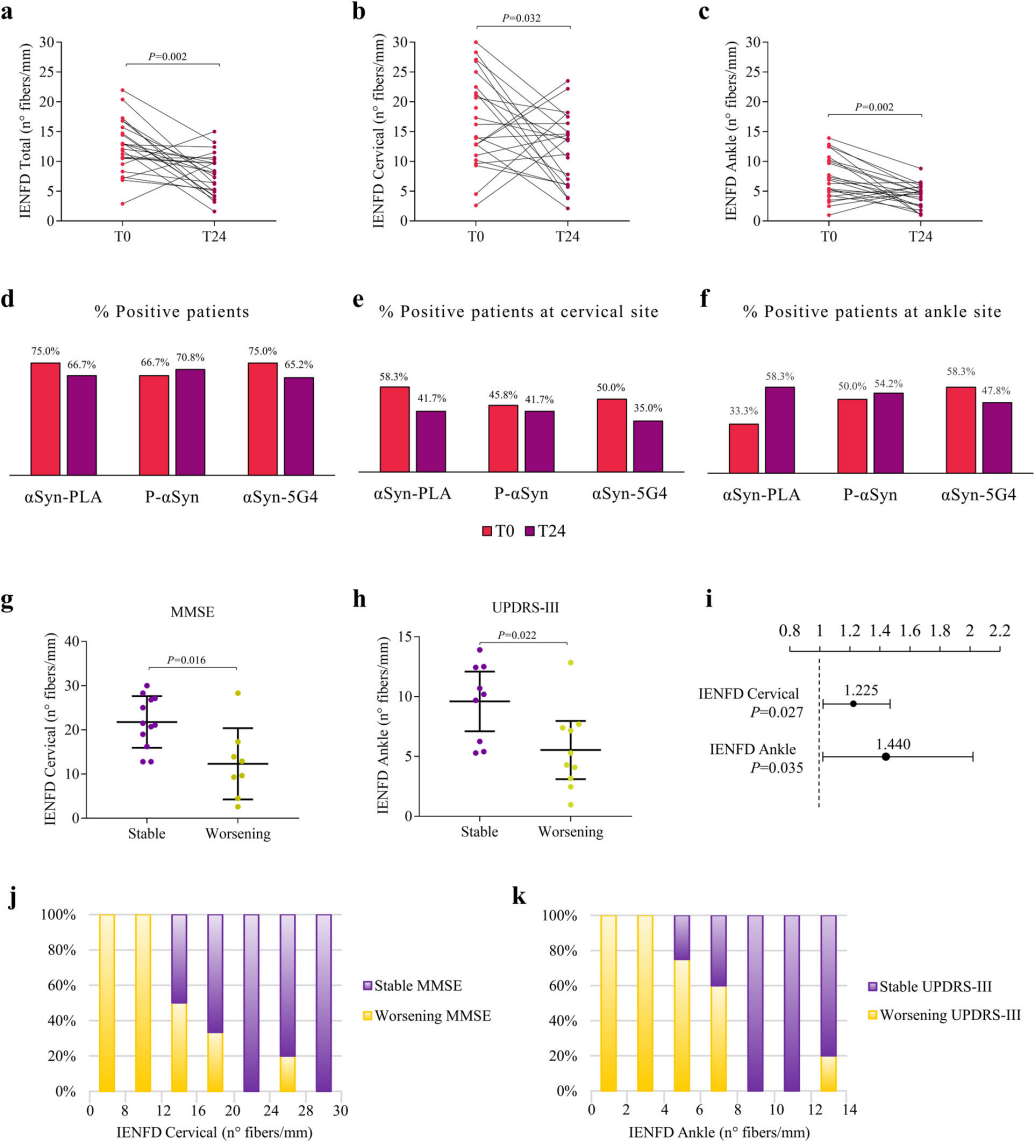
PD patients present a progression of denervation not of pathological αSyn at T24 and IENFD predicts cognitive and motor decline. **a** After 24 months PD patients showed a significant progression of intraepidermal denervation, **b**, **c** at both anatomical sites. **d**–**f** No significant differences were observed in the percentage of positivity for αSyn-PLA, P-αSyn, and αSyn-5G4 but a slight reduction for all markers at the cervical site and an increase of αSyn-PLA in the ankle are noted. **g** Patients with a worsening of MMSE scale at T24 showed a lower cervical IENFD at T0; **h** while a lower ankle IENFD was present at T0 in patients with a progressive motor impairment at MDS-UPDRS-III scale. **i** Univariate logistic regression analysis showed an association between cervical IENFD and MMSE, and between ankle IENFD and MDS-UPDRS-III; ODD ratio and *P* value are reported. **j**, **k** Percentage of patients with cognitive and motor progression at T24 are represented according to respectively cervical and ankle IENFD at T0.

The comparison between subjects with PD at time point 0 (T0) and after 24 months (T24), showed a significant reduction in total IENFD, ankle IENFD, and cervical IENFD (Fig. 5a–c). No differences in the percentage of patients positive for αSyn-PLA, P-αSyn, and αSyn-5G4 were detected (Fig. 5d–f), but a slight reduction for all markers at the cervical site and an increase of αSyn-PLA signal in the ankle were noted. No significant changes in clinical scales and LEDD were detected (Supp. Table 7).

### IENFD is a progression marker for PD

Comparisons of skin biopsy parameters at T0 according to the clinical state at T24 demonstrated that: (1) lower cervical IENFD was measured in those presenting a progression of cognitive decline at the MMSE scale and (2) lower ankle IENFD was observed in the group presenting a progression of motor impairment at the MDS-UPDRS-III scale (Fig. 5g, h). These results were confirmed by logistic regression, showing that low cervical IENFD was associated with an increased risk of developing a cognitive decline, while low ankle IENFD was associated with an increased risk of developing motor impairment at T24, after adjusting for age and LEDD (Fig. 5i–k).

### A skin biopsy-derived compound marker stratified subjects according to the clinical diagnosis

**Fig. 6:**
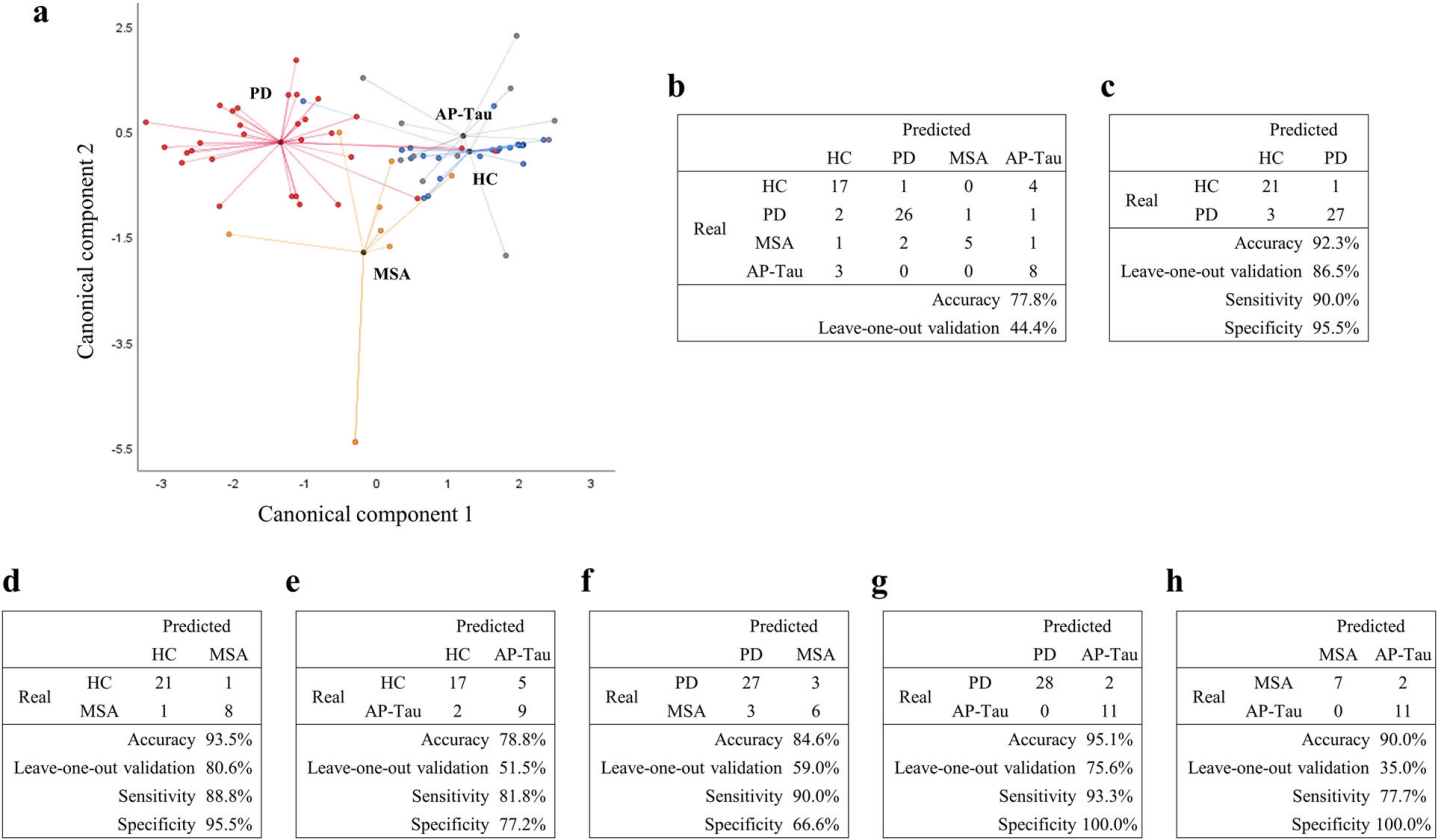
Skin biopsy-derived compound marker allows stratification of patients according to the diagnosis. **a** Canonical plot showing patients according to the diagnosis. The model was built considering the presence/absence of αSyn-PLA, P-αSyn, αSyn-5G4, and intraepidermal nerve fiber density (IENFD) at both anatomical sites. The axes of the plot (canonical components 1 and 2) were calculated from weighted linear combinations of variables to maximize the separation between the four groups. Each subject is represented by a point. **b**–**h** Confusion matrix reporting real and predicted diagnosis, accuracy, internal validation by the leave-one-out algorithm, sensitivity, and specificity is reported for all groups and pairwise comparisons.

A linear discriminant analysis model based on the IENFD and the expression of αSyn-PLA, P-αSyn, and αSyn-5G4 at both sites at T0, allowed the separation of subjects according to their clinical diagnosis (Fig. 6a). Patients were discriminated with 77.8% of accuracy: 26 out of 30 PD, and five out of nine MSA were correctly diagnosed, while the discrimination between HC and AP-Tau was more challenging (Fig. 6b). Subsequently, pairwise comparisons were performed (Fig. 6c–h), showing high sensitivity (77.7–93.3%) and specificity (66.6–100.0%) in the discrimination between two groups at the time.

## Discussion

We demonstrated that αSyn oligomers, detected in skin biopsy by PLA, are significantly more expressed in PD and MSA patients than in HC and AP-Tau and they yield a high diagnostic performance for synucleinopathies. Furthermore, we documented that significant denervation in both cervical and ankle sites occurs after two years of follow-up in PD patients and that low IENFD at T0 is associated with progression of disease at cognitive and motor scales.

The observation of αSyn oligomers in skin peripheral nerves is in accordance with previous studies in the postmortem brain of PD patients demonstrating αSyn-PLA signal in neuroanatomical regions usually mildly affected by pathology and in the absence of Lewy bodies^25^. These results underline how the αSyn-PLA technique specifically detects oligomers, which are supposedly involved at early phases of pathology, preceding the formation of insoluble aggregates in the brain. Notably, also in postmortem brains of MSA, where the pathological hallmark is represented by glial cytoplasmatic inclusions of αSyn, PLA methodology detected a widespread distribution of αSyn oligomers in neurons, as well as in oligodendrocytes^27^. Thus, albeit different molecular mechanisms of disease preferentially affecting glial cells are supposed to be implied in MSA versus PD, our results of overexpression of oligomeric αSyn in peripheral nerves in both groups are not surprising. Similarly, to what has been demonstrated in brains^27^, we observed larger colocalization areas of PLA signal within nerves in MSA than in PD, suggesting a larger amount of oligomeric αSyn in MSA, possibly reflecting different αSyn strains. The finding of the positive αSyn-PLA signal also in the HC group, even if in a nonsignificant low rate (10–20% depending on considering one or two sites), deserves consideration. We observed it mainly at the ankle, inside nerves, and never in association with fragmented, degenerating axons (dotted signal), differently from synucleinopathies. The presence of incidental αSyn pathology has been detected in brain autopsies of 10–20% of subjects >70 years, without neurological diseases^30^. Roberts et al. described a diffuse kind of αSyn-PLA signal in HC too, mainly located in the neuropil around neurons and less frequently in the white matter of brain areas usually affected by PD pathology^25^. The neuropil is mainly composed of unmyelinated axons, dendrites, and synapsis, which are very similar to the composition of small fiber nerves, either unmyelinated C fiber or small myelinated Aδ, present in the skin. Indeed, aggregates of αSyn, are preferentially formed in projection neurons with very long, thin unmyelinated axons, which are more susceptible to axonal transport deficit, energetic/metabolic stress, mitochondrial failure, and lack the trophic support of glial cells^31^. Finally, Mazzetti et al. detected a nonsignificant quote of αSyn-PLA signal in skin autonomic nerves of HC^29^. Thus, αSyn oligomers in HC may represent aberrant oligomerization of αSyn at the synapses or axonal transport of pathological species as a very early subclinical event that may become more relevant with aging and/or external toxic events in a susceptible population. However, our study cohort is mainly composed of established PD with long disease duration, and larger studies including prodromal phases of disease using αSyn-PLA technology in skin biopsies are warranted.

The anatomical site providing the best differentiation between PD vs. HC and AP-Tau is the cervical one while both anatomical sites are required to differentiate HC and AP-Tau from MSA, in which a higher presence of oligomeric αSyn at the ankle rather than at the cervical site is shown, differently from PD. This is in line with previous observations of a distal-to-proximal gradient of αSyn aggregates in MSA^32^, possibly consequent of a different spreading of αSyn oligomers, or because different αSyn strains have been postulated in MSA versus PD^33^.

The dermal autonomic structures presenting most of the PLA signal are SG in line with Mazzetti et al.^29^ followed by MAP. This result is in accordance with previous work showing P-αSyn mainly in SG of PD patients^34^. We found a similar major distribution of oligomeric αSyn in SG also in MSA, while the distribution of P-αSyn in autonomic or somatosensory nerve fibers in this group is more debated in the literature and some authors have shown a predominance of P-αSyn in somatosensory nerve fibers instead of autonomic ones^12^. However, while αSyn oligomers are small aggregates preceding the formation of fibrils in the early stages of pathology, P-αSyn accumulates in Lewy bodies, and yet it is still debated whether phosphorylation at Serine 129 enhances or suppresses αSyn aggregation and toxicity^35^, as this could be a later event in disease progression. Thus, αSyn-PLA and P-αSyn are probably markers of different stages of the disease. A recent study focused on αSyn content in MAP found that patients with Lewy-body neurogenic orthostatic hypotension displayed higher content of αSyn in sympathetic noradrenergic nerves than MSA^36^. Thus, based on the selection of different protein targets and different anatomical sites, skin biopsy analysis can distinguish between PD and MSA.

The comparative analysis of the diagnostic performance of αSyn-PLA vs. P-αSyn and 5G4 in discriminating PD from HC and AP-Tau has shown that αSyn-PLA displays the best accuracy when considering both locations and the cervical site alone. This result can also be explained by the fact that by detecting oligomers, the early species in the aggregation pathway, PLA can bear more sensitivity to early phases of disease than P-αSyn. However, P-αSyn and 5G4, possible markers of later phases of the disease, performed better when considering the ankle site alone. Thus, the choice of pathological αSyn marker and the site of skin biopsy influences the diagnostic performance importantly and can help to understand the temporal dynamics of α αSyn seeding and spreading in the peripheral nervous system during the course of the disease. Of note, αSyn-PLA was the only technique yielding discrimination between MSA and HC. These are remarkable results for αSyn-PLA considering that a very recent study has shown that P-αSyn detection in the skin has a higher diagnostic accuracy for synucleinopathies than real-time quaking-induced conversion (RT-QuIC) in the skin, a sensible and accurate technology to measure prion-like self-aggregation of pathological αSyn^37^. However, our results may be partially influenced by methodological issues, since αSyn-PLA signal was analyzed by confocal microscopy while P-αSyn and 5G4 were analyzed by fluorescence microscopy so that a better resolution was guaranteed in the first condition. It should be taken into account that in both cases the interpretation of immunofluorescence findings is a complex process and requires a trained pathologist.

We further confirmed the presence of a small fiber neuropathy in PD and MSA, as observed in previous studies with skin biopsy^14,22,38^. The neuropathy is not related to levodopa therapy or vitamin B12 deficiency, and it is very likely secondary to the neurodegeneration process^39,40^. More importantly, we showed that denervation progresses in a significant way with disease duration, even in a relatively short period of time (2 years), while no significant changes in clinical scales or in pathological αSyn amounts were detectable in this study. Furthermore, we demonstrated that a lower ankle IENFD at T0 was associated with a progression of motor impairment at the MDS-UPDRS-III scale and that a lower cervical IENFD was associated with a progression of cognitive decline at MMSE, independently from age and LEDD. These findings are in line with brain pathology studies showing that clinical symptoms in PD align with neuronal degeneration rather than with the αSyn aggregation burden^41^. Thus, the relevance of IENFD as a progression marker in PD is worthy of further investigations in larger studies, because if the clinical significance of IENFD changes is confirmed, skin denervation could be considered as a proxy measure of neurodegenerative events occurring in the brain and possibly an ancillary biomarker in clinical trials. Moreover, the measure of IENFD by skin biopsy bears the advantage of being easily and rapidly performed at low cost, as well as being extensively studied and standardized in the last twenty years for the diagnosis of small fiber neuropathy^42,43^. Nevertheless, an important limitation of the current study is that the most severely affected patients of the cohort were lost at follow-up, potentially limiting the ability to evaluate more aggressive forms with higher progression rates.

Of note, a compound marker based on all three markers for pathological αSyn and IENFD was able to stratify patients with high accuracy according to the diagnosis and to discriminate between PD and MSA (84.6%), which is a major challenge in clinical practice. In conclusion, skin biopsy represents a minimally-invasive, easily-accessible, and repeatable source of biomarkers for PD in vivo: multiple target epitopes of proteins involved in the pathogenesis of the disease are detectable and it allows the quantification of small fiber neuropathy through IENFD, which represents a promising prognostic marker. A thorough evaluation of multiple markers bearing complementary information and of small fiber pathology by skin biopsy is advisable not only for the diagnosis of PD in routine clinical practice but also for the future development of more adequate biomarkers as surrogate endpoints in pharmacological clinical trials for PD.

## Methods

### Patients’ recruitments

Thirty patients with idiopathic PD, 22 age and sex-matched HC, and 23 AP were prospectively recruited from the movement disorders outpatient clinic at the Neurocenter of Southern Switzerland in Lugano, from July 2015 to January 2021 as part of the NSIPD001 study^14^. Inclusion criteria for PD were a clinical diagnosis according to the UK Brain Bank diagnostic criteria^44^, disease duration of at least 2 years, no family history, and no major cognitive impairment or major autonomic dysfunction symptoms in the history. HC subjects were recruited among patients’ partners and hospital staff without any known pathology. AP group included 12 patients with probable MSA and 11 patients with possible AP-Tau, among which eight with probable progressive supranuclear palsy (PSP) and three with possible corticobasal degeneration (CBD). Inclusion criteria for AP were based on published diagnostic criteria for MSA^45^, PSP^46^, and CBD^47^. Exclusion criteria were co-morbidities causing peripheral polyneuropathies (diabetes, renal failure, thyroid pathology, vitamin B12 deficiency, HIV and HCV infection, Lyme disease, syphilis, acute or chronic inflammatory diseases) and tumors. After 2 years (T24) PD and AP patients underwent a follow-up clinical evaluation and a second skin biopsy (Fig.1).

The Cantonal Ethics Committee approved the study protocol, and all enrolled subjects gave written informed consent to the study.

### Clinical assessment

Disease severity was determined by the Hoehn and Yahr Scale (H&Y)^48^ and the Movement Disorder Society Unified Parkinson’s Disease Rating Scale (MDS-UPDRS)^49^. Cognitive impairment was assessed with the Mini-Mental State Examination (MMSE)^50^ and the Montreal Cognitive Assessment (MoCA)^51^. Symptoms of autonomic dysfunction were rated with the Composite Autonomic Symptom Score 31 (COMPASS-31)^52^. Mood disorders were screened with the Beck depression inventory-II (BDI-II)^53^, while REM sleep behavior disorder (RBD) with the RBD screening questionnaire;^54^ olfactory function was tested with the Sniffin’ Sticks Smell test (Burghart Messtechnik GmbH, Wedel, Germany). The levodopa equivalent daily dose (LEDD) was calculated for all PD and AP patients^55^.

### Skin biopsy

A 3-mm punch skin biopsy was performed on the clinically more affected side, as previously described^14,56^. Each subject underwent skin collection at two anatomical sites, namely at the C8 dermatomal level (cervical) and at the distal leg, about 10 cm above the lateral malleolus (ankle). Skin samples were fixed overnight at 4 °C in Paraformaldehyde-lysine-periodate (PLP) 2% fixative. The day after, skin samples were frozen and cut with a cryotome to obtain 50-μm-thin tissue sections for free-floating immunofluorescence analysis^14,42^. All the immunofluorescence staining were manually performed by an operator blind to the clinical diagnosis

### Intraepidermal nerve fiber density

Three non-consecutive 50-μm-thin tissue sections per localization per patient were stained with the antibody against protein gene product 9.5 (PGP9.5; Abcam, Cambridge UK, 1:1000)^56^. Cell nuclei were counterstained with 4′,6-diamidino-2-phenylindole (DAPI, Sigma Aldrich, Saint Louis USA, 1:5000). According to published standard protocols^42,57^, PGP9.5 positive nerve fibers, crossing the dermal-epidermal junction, were counted to determine the linear IENFD. The length of the section was measured and IENFD was obtained by dividing the number of fibers by the length of the section and expressed as “number of fibers/mm”. IENFD was determined at both ankle and cervical sites and total IENFD was calculated as the mean of nerve densities at both localizations.

### Direct proximity ligation assay (PLA)

Direct PLA was performed with the Duolink PLA kit (Duolink Kit Sigma Aldrich, Saint Louis USA). Following the manufacturer’s instructions, the oligonucleotides probes MINUS and PLUS were separately conjugated to the mouse monoclonal αSyn antibody with blocking activity (anti-αSyn 211, 1 mg/mL, Abcam, Cambridge, UK)^25^. Three non-consecutive 50-μm-thin tissue sections per localization per patient were washed in TBS and then incubated for 2 h at room temperature in a freshly made blocking solution (4% NGS, 1% Triton in TBS). Sections were incubated with αSyn-PLUS and αSyn-MINUS probes (1:100 in PLA diluent) and rabbit anti-PGP9.5 antibody (Abcam, Cambridge, UK, 1:1000) for 1 h at 37 °C and then overnight at room temperature.

The day after, the amplification reaction was performed by serial incubation with (1) ligase enzyme diluted 1:40 in ligation buffer (Duolink kit, Sigma Aldrich, Saint Louis USA) for 1 h at 37 °C; (2) polymerase enzyme diluted 1:80 in the Amplification buffer (Duolink kit, Sigma Aldrich, Saint Louis USA) and secondary antibodies AlexaFluor488 goat anti-rabbit (ThermoFisher Scientific, Waltham USA, 1:700) for 2 h at 37 °C. Finally, cell nuclei were counterstained with DAPI (1:5000 in PBS for 4 min) and section slides were mounted with Vectashield (Vector Laboratories, Burlingame, USA).

The specificity of the assay for αSyn-PLA was first assessed in paraffined brain sections from PD and HC (Supp. Fig. 1). Serial negative controls without oligonucleotide probes and amplification reaction enzymes were also conducted (Supp. Fig. 1).

### Confocal microscope analysis of αSyn-PLA

To increase the specificity of the analysis we considered only αSyn-PLA signal within the nerves which colocalized with the axonal marker PGP9.5 (colocalization signal) or which was located in proximity to degenerated nerve fibers, defined by axonal swellings and fragmentations or interposed between nerve fiber breakpoints (dotted signal) (Fig. 2a).

Skin sections were first rated under an inverted fluorescence microscope (Nikon Eclipse Ti-E, Tokyo, Japan). For each section, both somatosensory and autonomic innervation were screened: intraepidermal nerve fibers, dermal nerve bundles, and autonomic structures (sweat glands [SG], muscle arrector pili [MAP], and vessels). All SG and MAP were analyzed by a Nikon confocal microscope using the NIS Elements 4.11.01 imaging software. Serial pictures, every 2 μm on the Z-axis, were taken using a 20X magnification. To avoid missing small amounts of signal within dermal nerve bundles, we also analyzed at least three nerve bundles that were randomly chosen at each anatomical site by confocal microscope. Every single stack of all the obtained pictures was analyzed with ImageJ software by two independent examiners, blinded to the clinical diagnosis. For each image, we reported the presence/absence of signal. Structures were defined as “positive” if showing αSyn-PLA. Anatomical sites were defined as “positive” when at least one positive structure was present. The subjects were defined as “positive” when they showed at least one positive site. Negative subjects did not present PLA signals at any site. To assess the anatomical distribution of PLA signal in each group, we also considered ankle and cervical sites separately.

### Quantification of αSyn-PLA

For each patient, the percentage of positive SG and MAP structures was calculated. For each structure, the number of colocalization points was counted and, if one subject showed signal in multiple structures and sites, the sum of the points was considered. Finally, we calculated the area occupied by αSyn-PLA signal normalized for the area of innervation. First, the stacks on the Z-axis were merged, then the area of the structure (DAPI), the area of innervation (PGP9.5), and the area of αSyn-PLA within nerves were measured with the “freehand selection” tool. The area of innervation was normalized for the area of the structure and then the percentage of the innervation area occupied by αSyn-PLA signal was measured.

### Phosphorylated alpha-synuclein and 5G4 immunofluorescence

Three non-consecutive 50-μm-thin tissue sections per localization per patient were incubated overnight with antibodies against PGP9.5 (1.1000, Abcam, Cambridge, UK), P-αSyn at serine 129 (1.1000, Wako Chemicals, Neuss, Germany) or 5G4 (1.400, Analytik Jena Life Science, Jena, Germany)^56^. The day after, sections were incubated with secondary antibody AlexaFluor488 and AlexaFluor594 (ThermoFisher Scientific, Waltham USA, 1:700), and counterstained with DAPI. All sections were analyzed at a fluorescence microscope by two independent raters blinded to the diagnosis. Colocalization of P-αSyn/5G4 and PGP9.5 was considered as positive signal as previously described^14^.

### Statistical analysis

Statistical analyses were performed with IBM SPSS Statistics 26.0 (IBM Corp. Released 2019. IBM SPSS Statistics for Windows, Version 26.0. Armonk, NY: IBM Corp). Variable distribution was assessed by the Kolmogorov–Smirnov test. One-way ANOVA test with post hoc Bonferroni’s test for multiple comparisons was used for normally distributed variables expressed as mean ± standard deviation. Kruskal–Wallis’ test was applied to non-normally distributed variables expressed as medians and interquartile range. *χ*^2^ or Fisher’s exact tests were used for categorical variables expressed as a percentage (%). Odds ratios (OR) were assessed by univariate logistic regression analysis, while the area under the curve (AUC) and the diagnostic performances of selected variables were obtained with receiver operating characteristics (ROC) curves analysis. For matched analysis (T0 vs. T24), non-normally distributed variables were analyzed by Wilcoxon pairs signed-rank test, and categorical variables by McNemar test. Associations between IEFND (cervical, ankle, and total) at T0 and worsening of clinical characteristics (MoCA, MMSE, MDS-UPDRS-III) at T24 were tested using logistic regression models adjusted by age and LEDD, with OR and 95% confidence intervals calculation (95% CI).

The linear discriminant analysis model was used to classify patients. Canonical components 1 and 2 were calculated from weighted linear combinations of variables (αSyn-PLA, P-αSyn, αSyn-5G4, and IENFD) to maximize the separation between groups.

### Reporting summary

Further information on research design is available in the Nature Research Reporting Summary linked to this article.

## Supplementary information

Supplementary Information

Reporting Summary

## Data Availability

The raw data that support the findings of this article are available on reasonable request to the corresponding author.

## References

[1] Spillantini MG, Goedert M (2000). The alpha-synucleinopathies: Parkinson’s disease, dementia with Lewy bodies, and multiple system atrophy. Ann. N. Y Acad. Sci..

[2] Krismer F, Wenning GK (2017). Multiple system atrophy: insights into a rare and debilitating movement disorder. Nat. Rev. Neurol..

[3] Klingelhoefer L, Reichmann H (2017). Parkinson’s disease as a multisystem disorder. J. Neural Transm..

[4] Kumaresan M, Khan S (2021). Spectrum of non-motor symptoms in Parkinson’s disease. Cureus.

[5] Beach TG (2010). Multi-organ distribution of phosphorylated alpha-synuclein histopathology in subjects with Lewy body disorders. Acta Neuropathol..

[6] Lee JM (2017). The search for a peripheral biopsy indicator of alpha-synuclein pathology for Parkinson disease. J. Neuropathol. Exp. Neurol..

[7] Shannon KM (2012). Alpha-synuclein in colonic submucosa in early untreated Parkinson’s disease. Mov. Disord..

[8] Irwin D, Saint-Hilaire M (2021). In vivo detection of underlying synucleinopathies: are we there yet?. Neurology.

[9] Stokholm MG, Danielsen EH, Hamilton-Dutoit SJ, Borghammer P (2016). Pathological alpha-synuclein in gastrointestinal tissues from prodromal Parkinson disease patients. Ann. Neurol..

[10] Del Tredici K, Hawkes CH, Ghebremedhin E, Braak H (2010). Lewy pathology in the submandibular gland of individuals with incidental Lewy body disease and sporadic Parkinson’s disease. Acta Neuropathol..

[11] Donadio V (2014). Skin nerve alpha-synuclein deposits: a biomarker for idiopathic Parkinson disease. Neurology.

[12] Doppler K (2015). Distinctive distribution of phospho-alpha-synuclein in dermal nerves in multiple system atrophy. Mov. Disord..

[13] Gibbons CH, Garcia J, Wang N, Shih LC, Freeman R (2016). The diagnostic discrimination of cutaneous alpha-synuclein deposition in Parkinson disease. Neurology.

[14] Melli G (2018). Cervical skin denervation associates with alpha-synuclein aggregates in Parkinson disease. Ann. Clin. Transl. Neurol..

[15] Iwanaga K (1999). Lewy body-type degeneration in cardiac plexus in Parkinson’s and incidental Lewy body diseases. Neurology.

[16] Kovacs GG (2014). Intracellular processing of disease-associated alpha-synuclein in the human brain suggests prion-like cell-to-cell spread. Neurobiol. Dis..

[17] Kovacs GG (2012). An antibody with high reactivity for disease-associated alpha-synuclein reveals extensive brain pathology. Acta Neuropathol..

[18] Kumar ST (2020). How specific are the conformation-specific alpha-synuclein antibodies? Characterization and validation of 16 alpha-synuclein conformation-specific antibodies using well-characterized preparations of alpha-synuclein monomers, fibrils and oligomers with distinct structures and morphology. Neurobiol. Dis..

[19] Iranzo, A. et al. alpha-Synuclein aggregates in labial salivary glands of idiopathic rapid eye movement sleep behavior disorder. *Sleep*10.1093/sleep/zsy101 (2018). 10.1093/sleep/zsy101 29790977

[20] Skorvanek M (2018). alpha-Synuclein antibody 5G4 identifies manifest and prodromal Parkinson’s disease in colonic mucosa. Mov. Disord..

[21] Tsukita K, Sakamaki-Tsukita H, Tanaka K, Suenaga T, Takahashi R (2019). Value of in vivo alpha-synuclein deposits in Parkinson’s disease: a systematic review and meta-analysis. Mov. Disord..

[22] Nolano M (2018). Small fiber pathology parallels disease progression in Parkinson disease: a longitudinal study. Acta Neuropathol..

[23] Kass-Iliyya L (2015). Small fiber neuropathy in Parkinson’s disease: a clinical, pathological and corneal confocal microscopy study. Parkinsonism Relat. Disord..

[24] Bellucci A, Fiorentini C, Zaltieri M, Missale C, Spano P (2014). The “in situ” proximity ligation assay to probe protein-protein interactions in intact tissues. Methods Mol. Biol..

[25] Roberts RF, Wade-Martins R, Alegre-Abarrategui J (2015). Direct visualization of alpha-synuclein oligomers reveals previously undetected pathology in Parkinson’s disease. Brain. Brain.

[26] Bengoa-Vergniory N, Roberts RF, Wade-Martins R, Alegre-Abarrategui J (2017). Alpha-synuclein oligomers: a new hope. Acta Neuropathol..

[27] Sekiya H (2019). Wide distribution of alpha-synuclein oligomers in multiple system atrophy brain detected by proximity ligation. Acta Neuropathol..

[28] Ruffmann C (2018). Detection of alpha-synuclein conformational variants from gastro-intestinal biopsy tissue as a potential biomarker for Parkinson’s disease. Neuropathol. Appl. Neurobiol..

[29] Mazzetti S (2020). alpha-Synuclein oligomers in skin biopsy of idiopathic and monozygotic twin patients with Parkinson’s disease. Brain.

[30] Beach TG (2009). Unified staging system for Lewy body disorders: correlation with nigrostriatal degeneration, cognitive impairment and motor dysfunction. Acta Neuropathol..

[31] Braak H, Rub U, Gai WP, Del Tredici K (2003). Idiopathic Parkinson’s disease: possible routes by which vulnerable neuronal types may be subject to neuroinvasion by an unknown pathogen. J. Neural Transm..

[32] Donadio V (2018). Skin alpha-synuclein deposits differ in clinical variants of synucleinopathy: an in vivo study. Sci. Rep..

[33] Peng C, Trojanowski JQ, Lee VM (2020). Protein transmission in neurodegenerative disease. Nat. Rev. Neurol..

[34] Doppler, K. Detection of dermal alpha-synuclein deposits as a biomarker for Parkinson’s disease. *J. Parkinsons Dis*. 10.3233/JPD-202489 (2021). 10.3233/JPD-202489 PMC8461714 33814464

[35] Oueslati A (2016). Implication of alpha-synuclein phosphorylation at S129 in synucleinopathies: what have we learned in the last decade?. J. Parkinsons Dis..

[36] Isonaka R, Gibbons CH, Wang N, Freeman R, Goldstein DS (2019). Association of innervation-adjusted alpha-synuclein in arrector pili muscles with cardiac noradrenergic deficiency in autonomic synucleinopathies. Clin. Auton. Res..

[37] Donadio V (2021). In vivo diagnosis of synucleinopathies: a comparative study of skin biopsy and RT-QuIC. Neurology.

[38] Donadio V (2020). Skin biopsy may help to distinguish multiple system atrophy-Parkinsonism from Parkinson’s disease with orthostatic hypotension. Mov. Disord..

[39] Che NN, Yang HQ (2020). Potential use of corneal confocal microscopy in the diagnosis of Parkinson’s disease associated neuropathy. Transl. Neurodegener..

[40] Cossu G, Melis M (2016). The peripheral nerve involvement in Parkinson disease: a multifaceted phenomenon. Parkinsonism Relat. Disord..

[41] Espay AJ (2020). Disease modification and biomarker development in Parkinson disease: revision or reconstruction?. Neurology.

[42] Lauria G (2005). EFNS guidelines on the use of skin biopsy in the diagnosis of peripheral neuropathy. Eur. J. Neurol..

[43] Lauria G (2010). European Federation of Neurological Societies/Peripheral Nerve Society Guideline on the use of skin biopsy in the diagnosis of small fiber neuropathy. Report of a joint task force of the European Federation of Neurological Societies and the Peripheral Nerve Society. Eur. J. Neurol..

[44] Hughes AJ, Daniel SE, Kilford L, Lees AJ (1992). Accuracy of clinical diagnosis of idiopathic Parkinson’s disease: a clinico-pathological study of 100 cases. J. Neurol. Neurosurg. Psychiatry.

[45] Gilman S (2008). Second consensus statement on the diagnosis of multiple system atrophy. Neurology.

[46] Hoglinger GU (2017). Clinical diagnosis of progressive supranuclear palsy: the movement disorder society criteria. Mov. Disord..

[47] Armstrong MJ (2013). Criteria for the diagnosis of corticobasal degeneration. Neurology.

[48] Hoehn MM, Yahr MD (1967). Parkinsonism: onset, progression and mortality. Neurology.

[49] Fahn, S. & Elton, R. UPDRS program members. In *Recent Developments in Parkinsons Disease* Vol. 2 (eds. Fahn, S., Marsden, C. D., Goldstein, M. & Calne, D. B.) (Macmillan Healthcare Information, 1987).

[50] Folstein MF, Folstein SE, McHugh PR (1975). “Mini-mental state”. A practical method for grading the cognitive state of patients for the clinician. J. Psychiatr. Res..

[51] Nasreddine ZS (2005). The Montreal cognitive assessment, MoCA: a brief screening tool for mild cognitive impairment. J. Am. Geriatr. Soc..

[52] Sletten DM, Suarez GA, Low PA, Mandrekar J, Singer W (2012). COMPASS 31: a refined and abbreviated composite autonomic symptom score. Mayo Clin. Proc..

[53] Beck, A. T., Steer, R. A. & Brown, G. K. *Manual for the Beck Depression Inventory-II*. (San Antonio, TX, Psychological Corporation, (1996).

[54] Stiasny-Kolster K (2007). The REM sleep behavior disorder screening questionnaire - A new diagnostic instrument. Mov. Disord..

[55] Tomlinson CL (2010). Systematic review of levodopa dose equivalency reporting in Parkinson’s disease. Mov. Disord..

[56] Vacchi, E., Pinton, S., Kaelin-Lang, A. & Melli, G. Targeting alpha synuclein aggregates in cutaneous peripheral nerve fibers by free-floating immunofluorescence assay. *J. Vis. Exp*. 10.3791/59558 (2019). 10.3791/59558 31305509

[57] Provitera V (2016). A multi-center, multinational age- and gender-adjusted normative dataset for immunofluorescent intraepidermal nerve fiber density at the distal leg. Eur. J. Neurol..

