# Supplementary material for: Alpha-synuclein oligomers and small nerve fiber pathology in skin are potential biomarkers of Parkinson’s disease
Source: NPJ Parkinsons Dis. 2021 Dec 20;7:119. doi: 10.1038/s41531-021-00262-y (PMC8688481; doi:10.1038/s41531-021-00262-y)
Supplement: Supplementary file 1 — Supplementary Information [file 41531_2021_262_MOESM1_ESM.pdf]

SUPPLEMENTARY MATERIAL

Oligomeric α-synuclein and small nerve fiber pathology in skin biopsy are biomarkers of Parkinson's disease: a longitudinal study

Elena Vacchi[1,2], Camilla Senese[1], Giacomo Chiaro[3], Giulio Disanto[3], Sandra Pinton[1], Sara Morandi[3], Ilaria Bertaina[3], Giovanni Bianco[3], Claudio Staedler[3], Salvatore Galati[2,3], Claudio Gobbi[2,3], Alain Kaelin-Lang[1,2,3,4], Giorgia Melli[1,2,3,*]

1. Laboratory for Biomedical Neurosciences, Neurocenter of Southern Switzerland, Ente Ospedaliero Cantonale, Lugano, Switzerland
2. Faculty of Biomedical Sciences, Università della Svizzera Italiana, Lugano, Switzerland
3. Neurology Department, Neurocenter of Southern Switzerland, Ente Ospedaliero Cantonale, Lugano, Switzerland
4. Department of Neurology, Inselspital, Bern University Hospital, University of Bern, Bern, Switzerland

*Corresponding author

Giorgia Melli, Tel: +41 (0)91 8116535; Fax: +41 (0)918116915; E-mail: giorgia.melli@eoc.ch

Summary:

Supplementary Table 1. Comparison of drop-out vs. not drop-out PD patients at T24

Supplementary Figure 1. αSyn-PLA technical controls

Supplementary Table 2. αSyn-PLA positivity

Supplementary Table 3. αSyn-PLA quantification

Supplementary Table 4. Comparison of PD patients with H&Y<3 vs H&Y≥3

Supplementary Table 5. αSyn-PLA positivity according to H&Y

Supplementary Table 6. Comparison of αSyn-PLA vs. P-αSyn vs. αSyn-5G4

Supplementary Table 7. Comparison of PD patients at T0 vsT24

Supplementary Table 1. Comparison of drop-out vs not drop-out PD patients at T24

**Variable**	**Drop-out** [n=6]	**Not drop-out** [n=24]	**Overall *P*-value**
Age (years)	74 ± 4	65 ± 12	0.158
Sex (ref. male)	33.3%	66.7%	0.153
Disease duration (years)	8.5 (3.8-20.5)	4.0 (2.8-7.3)	0.078
H&Y	3.0 (2.0-3.0)	2.0 (1.0-2.8)	**0.050**
MDS-UPDRS-I	6.0 (3.5-18.0)	3.5 (2.0-6.0)	0.113
MDS-UPDRS-II	6.0 (2.0-24.0)	5.5 (2.0-9.3)	0.739
MDS-UPDRS-III	22.0 (18.5-33.3)	14.0 (8.0-20.0)	**0.031**
MDS-UPDRS Total	32.0 (26.0-80.5)	23.0 (12.0-33.0)	0.086
COMPASS-31 Total	10.8 (5.8-33.8)	17.8 (0.0-24.1)	0.494
BDI-II	6.0 (3.0-7.0)	5.0 (3.0-9.0)	0.862
MMSE	29.0 (26.0-29.5)	30.0 (29.0-30.0)	0.082
MoCA	27.0 (18.0-29.5)	28.0 (25.0-29.0)	0.684
Olfactory test	5.0 (4.0-6.0)	7.0 (4.0-9.0)	0.469
RBD questionnaire	4.0 (1.0-6.5)	3.0 (1.0-5.0)	0.581
LEDD (mg)	682.5 (235.0-940.5)	375.0 (180.0-688.0)	0.302
IENFD total (N°fibers/mm)	10.2 (5.4-10.8)	12.5 (9.8-15.5)	**0.038**
IENFD cervical (N°fibers/mm)	15.9 (10.4-17.5)	16.8 (11.5-23.4)	0.127
IENFD ankle (N°fibers/mm)	4.5 (3.9-5.7)	6.9 (4.3-10.2)	0.594

Abbreviations: H&Y (Hoehn and Yahr scale), MDS-UPDRS (Movement Disorder Society-Unified Parkinson's Disease Rating Scale) I, II, III and total, composite autonomic symptom scale 31 (COMPASS-31); BDI-II (Beck Depression Inventory II), MMSE (Mini-Mental State Examination), MoCA (Montreal Cognitive Assessment), RBD (Rem Behaviour Disorder), LEDD (LevoDopa equivalent Dose), IENFD (intraepidermal nerve fiber density). P-values < 0.05 were considered significant and shown in bold.

Supplementary Figure 1. αSyn-PLA technical controls

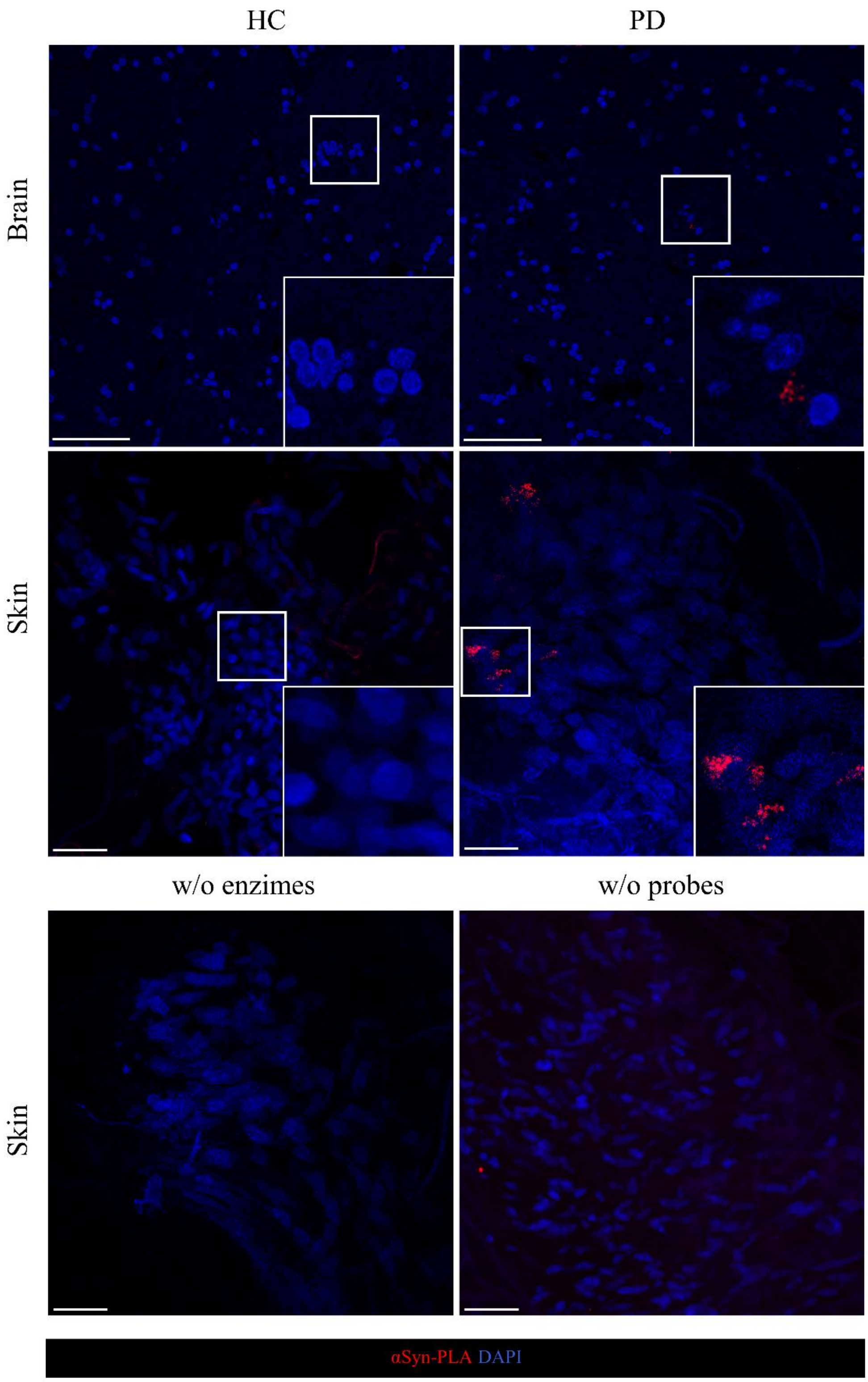

αSyn-PLA staining in basal ganglia and mid-brain region obtained from post-mortem human brains of HC and PD patients respectively, compared to the signal obtained in cervical skin. In the bottom panel, negative controls are shown without amplification reaction enzymes or without oligonucleotide probes.

Supplementary Table 2. αSyn-PLA positivity

	Type of signal	HC [n=22]	PD [n=30]	MSA [n=9]	AP-Tau [n=11]	Overall *P*-value	*Pairwise Comparisons* HC *vs.* PD	HC *vs.* MSA	HC vs. Tau	PD vs. MSA	PD vs. Tau	MSA vs. Tau
Positivity at Cervical or Ankle	αSyn-PLA	22.7%	80.0%	66.7%	18.2%	**<0.000**	**0.000**	**0.029**	0.571	0.338	**0.001**	**0.040**
	Colocalization	22.7%	76.7%	66.7%	18.2%	**<0.000**	**0.000**	**0.029**	0.571	0.419	**0.001**	**0.040**
	Dotted	0.0%	26.7%	22.2%	0.0%	**0.019**	**0.008**	0.077	-	0.581	0.061	0.198
	SG	18.2%	50.0%	66.7%	9.1%	**0.005**	**0.018**	**0.015**	0.450	0.312	**0.018**	**0.012**
	MAP	4.5%	20.0%	22.2%	0.0%	0.160	-	-	-	-	-	-
Positivity at Cervical	αSyn-PLA	9.1%	56.7%	33.3%	0.0%	**0.001**	**0.000**	0.219	0.282	0.199	**0.001**	0.074
	Colocalization	9.1%	53.3%	33.3%	0.0%	**0.001**	**0.001**	0.131	0.437	0.252	**0.001**	0.074
	Dotted	0.0%	20.0%	0.0%	0.0%	**0.027**	**0.029**	-	-	0.182	0.132	-
	SG	4.5%	33.3%	33.3%	0.0%	**0.014**	**0.012**	0.063	0.667	0.647	**0.027**	0.074
	MAP	4.5%	16.7%	11.1%	0.0%	0.318	-	-	-	-	-	-
Positivity at Ankle	αSyn-PLA	13.6%	40.0%	44.4%	18.2%	0.115	-	-	-	-	-	-
	Colocalization	13.6%	40.0%	44.4%	18.2%	0.115	-	-	-	-	-	-
	Dotted	0.0%	6.7%	22.2%	0.0%	0.079	-	-	-	-	-	-
	SG	13.6%	30.0%	44.4%	9.0%	0.148	-	-	-	-	-	-
	MAP	0.0%	3.3%	22.2%	0.0%	**0.032**	0.577	0.077	-	0.127	0.732	0.198

The table reports for each group the percentage of subjects positive to αSyn-PLA, considering type of signal (co-localization and dotted signal), autonomic structure (sweat glands [SG] or muscle arrector pili [MAP]) and anatomical sites (cervical and ankle or both). *P*-values < 0.05 were considered significant and showed in bold. Abbreviation: HC= healthy control, PD= Parkinson's Disease, MSA= multiple system atrophy, AP-Tau= atypical parkinsonism with tauopathy.

Supplementary Table 3. αSyn-PLA quantification

	**Type of signal**	**HC** [n=22]	**PD** [n=30]	**MSA** [n=9]	**AP-Tau** [n=11]	**Overall *P*-value**	***Pairwise Comparisons***					
							HC *vs.* PD	HC *vs.* MSA	HC vs. Tau	PD vs. MSA	PD vs. Tau	MSA vs. Tau
Both Localisations	N° tot αSyn-PLA	4.0 (1.5-5.5)	4.0 (2.0-10.5)	11.5 (5.5-19.0)	2.5 (2.0-3.0)	0.122	-	-	-	-	-	-
	N° co-localization points	4.0 (1.5-5.5)	3.0 (2.0-6.0)	5.5 (3.5-19.0)	2.5 (2.0-3.0)	0.361	-	-	-	-	-	-
	N° dotted points	0.0 (0.0-0.0)	4.5 (3.3-12.8)	8.0 (4.0-12.0)	0.0 (0.0-0.0)	0.792	-	-	-	-	-	-
	% Area Tot	9.0%	5.0%	23.0%	4.0%	**0.008**	0.684	**0.030**	0.381	**0.000**	0.480	0.071
	% Area SG	6.0%	6.0%	15.0%	6.0%	0.099	-	-	-	-	-	-
	% Area MAP	13.0%	7.0%	22.0%	0.0%	0.072	-	-	-	-	-	-
Cervical	N° tot αSyn-PLA	3.5 (1.0-6.0)	3.5 (1.0-8.8)	7.0 (4.0-13.0)	0.0 (0.0-0.0)	0.344	-	-	-	-	-	-
	N° co-localization points	3.5 (1.0-6.0)	2.0 (1.0-5.0)	7.0 (4.0-13.0)	0.0 (0.0-0.0)	0.132	-	-	-	-	-	-
	N° dotted points	0.0 (0.0-0.0)	4.5 (3.8-9.5)	0.0 (0.0-0.0)	0.0 (0.0-0.0)	-	-	-	-	-	-	-
	% Area Tot	11.0%	5.0%	22.0%	0.0%	**0.020**	0.132	0.400	-	**0.010**	-	-
	% Area SG	9.0%	4.0%	13.0%	0.0%	0.133	-	-	-	-	-	-
	% Area MAP	13.0%	7.0%	17.0%	0.0%	0.145	-	-	-	-	-	-
Ankle	N° tot αSyn-PLA	4.0 (2.0-5.0)	4.0 (1.3-16.5)	15.5 (8.3-16.0)	2.5 (2.0-3.0)	0.244	-	-	-	-	-	-
	N° co-localization points	4.0 (2.0-5.0)	3.0 (1.3-7.8)	9.5 (2.5-15.8)	2.5 (2.0-3.0)	0.632	-	-	-	-	-	-
	N° dotted points	0.0 (0.0-0.0)	8.5 (2.0-15.0)	8.0 (4.0-12.0)	0.0 (0.0-0.0)	1.000	-	-	-	-	-	-
	% Area Tot	3.0%	7.0%	20.0%	4.0%	0.064	-	-	-	-	-	-
	% Area SG	3.0%	6.0%	13.0%	6.0%	0.560	-	-	-	-	-	-
	% Area MAP	0.0%	3.0%	14.0%	0.0%	-	-	-	-	-	-	-

For each group, the table reports: the number of αSyn-PLA points considering co-localization and dotted signal together (N° tot αSyn-PLA) or separately, and the percentage of the innervation area occupied by αSyn-PLA signal, considering SG and MAP together (Area Tot) or separately. Data are reported according to the anatomical site. *P*-values < 0.05 were considered significant and showed in bold. Abbreviation: HC= healthy control, PD= Parkinson's Disease, MSA= multiple system atrophy, AP-Tau= atypical parkinsonism with tauopathy, SG=sweat gland, MAP =muscle arrector pili.

Supplementary Table 4. Comparison of PD patients with H&Y<3 vs. H&Y≥3

**Variable**	**H&Y<3** [n=20]	**H&Y≥3** [n=10]	**Overall *P*-value**
Age (years)	61 ± 9	77 ± 6	**<0.000**
Sex (ref. male)	55.0%	70.0%	0.350
Disease duration (years)	4.5 (3.7-9.0)	3.0 (2.0-7.3)	0.265
MDS-UPDRS-I	4.0 (2.0-6.0)	5.0 (2.3-8.3)	0.549
MDS-UPDRS-II	6.0 (2.0-10.0)	5.0 (2.5-14.3)	0.897
MDS-UPDRS-III	14.0 (8.0-19.5)	20.0 (17.5-28.0)	**0.023**
MDS-UPDRS Total	25.0 (12.0-33.0)	31.5 (21.5-51.5)	0.180
COMPASS-31 Total	18.4 (0.0-30.8)	8.6 (3.6-21.1)	0.914
BDI-II	5.0 (2.3-8.0)	6.0 (3.8-12.3)	0.381
MMSE	30.0 (30.0-30.0)	29.0 (26.5-29.5)	**0.028**
MoCA	28.0 (25.3-29)	26.0 (17.5-27.0)	0.070
Olfactory test	7.0 (4.0-9.0)	4.5 (4.0-5.0)	0.308
RBD questionnaire	3.0 (2.0-5.0)	2.0 (1.0-5.5)	0.594
LEDD (mg)	350.0 (100.0-688.0)	529.0 (323.5-871.3)	0.211
IENFD total (Fibers/mm)	11.5 (7.9-14.6)	10.9 (9.6-13.7)	0.880
IENFD cervical (Fibers/mm)	16.2 (10.2-22.5)	15.9 (13.1-22.2)	0.946
IENFD ankle (Fibers/mm)	6.3 (4.5-10.7)	5.1 (3.9-8.3)	0.456

Clinical characteristics of patients with Parkinson's disease (PD) according to Hoehn and Yahr scale (H&Y). Abbreviations: MDS-UPDRS (Movement Disorder Society-Unified Parkinson's Disease Rating Scale) I, II, III and total, composite autonomic symptom scale 31 (COMPASS-31); BDI-II (Beck Depression Inventory II), MMSE (Mini-Mental State Examination), MoCA (Montreal Cognitive Assessment), RBD (Rem Behaviour Disorder), LEDD (LevoDopa equivalent Dose), IENFD (intraepidermal nerve fiber density). P-values < 0.05 were considered significant and shown in bold.

Supplementary Table 5. αSyn-PLA positivity according to H&Y

**H&Y**	**Type of signal**	**H&Y<3** [n=20]	**H&Y≥3** [n=10]	**Overall *P*-value**
Both Localizations	αSyn-PLA	70.0%	100.0%	0.065
	SG	45.0%	60.0%	0.350
	MAP	15.0%	30.0%	0.306
	N° tot αSyn-PLA	5.0 (2.8-13.0)	4.0 (1.8-9.5)	0.585
	% Area Tot	7.0%	6.0%	0.975
Cervical	αSyn-PLA	60.0%	50.0%	0.446
	SG	40.0%	20.0%	0.251
	MAP	15.0%	20.0%	0.551
	N° tot αSyn-PLA	3.5 (1.3-8.8)	3.5 (1.0-9.8)	0.862
	% Area Tot	4.0%	7.0%	0.412
Ankle	αSyn-PLA	25.0%	70.0%	**0.024**
	SG	20.0%	50.0%	0.104
	MAP	0.0%	10.0%	0.333
	N° tot αSyn-PLA	19.0 (3.0-21.0)	2.0 (1.0-4.0)	**0.048**
	% Area Tot	8.0%	6.0%	0.268

PD patients were split in two groups according to Hoehn and Yahr scale (H&Y). The table reports for each group the percentage of subjects positive to αSyn-PLA considering autonomic structure, and αSyn-PLA quantification. Data are shown according to the anatomical site. *P*-values < 0.05 were considered significant and showed in bold.

Supplementary Table 6. Comparison of αSyn-PLA vs. P-αSyn vs. αSyn-5G4

	Type of signal	HC [n=22]	PD [n=30]	MSA [n=9]	AP-Tau [n=11]	Overall *P*-value	*Pairwise Comparisons* HC *vs.* PD	HC *vs.* MSA	HC vs. Tau	PD vs. MSA	PD vs. Tau	MSA vs. Tau
Positivity at cervical or ankle	αSyn-PLA	22.7%	80.0%	66.7%	18.2%	**<0.000**	**0.000**	**0.029**	0.571	0.338	**0.001**	**0.040**
	P-αSyn	20.0%	71.0%	44.4%	27.3%	**0.002**	**0.000**	0.180	0.484	0.142	**0.015**	0.370
	αSyn-5G4	20.0%	74.2%	55.6%	18.2%	**0.000**	**0.000**	0.071	0.646	0.249	**0.002**	0.102
Positivity at Cervical	αSyn-PLA	9.1%	56.7%	33.3%	0.0%	**0.001**	**0.000**	0.219	0.282	0.199	**0.001**	0.074
	P-αSyn	10.0%	48.4%	22.2%	20.0%	**0.020**	**0.004**	0.364	0.447	0.155	0.079	0.625
	αSyn-5G4	10.0%	54.8%	33.3%	18.2%	**0.006**	**0.001**	0.157	0.447	0.225	**0.038**	0.396
Positivity at Ankle	αSyn-PLA	13.6%	40.0%	44.4%	18.2%	0.115	-	-	-	-	-	-
	P-αSyn	20.0%	54.8%	33.3%	9.1%	**0.014**	**0.014**	0.369	0.405	0.225	**0.009**	0.217
	αSyn-5G4	15.8%	54.8%	37.5%	9.1%	**0.008**	**0.006**	0.227	0.530	0.317	**0.009**	0.177

The table reports for each group the percentage of subjects positive to αSyn-PLA, P-αSyn and αSyn-5G4. Data are shown according to the anatomical site. *P*-values < 0.05 were considered significant and showed in bold. Abbreviation: HC= healthy control, PD= Parkinson's Disease, MSA= multiple system atrophy, AP-Tau= atypical parkinsonism with tauopathy.

Supplementary Table 7. Comparison of PD patients at T0 vsT24

**Variable**	**T0** [n=24]	**T24** [n=24]	**Overall *P*-value**
H&Y	2.0 (1.0-2.8)	2.0 (2.0-2.6)	0.217
MDS-UPDRS-I	3.5 (2.0-6.0)	6.0 (2.0-7.0)	0.229
MDS-UPDRS-II	5.5 (2.0-9.3)	7.0 (5.0-9.0)	0.582
MDS-UPDRS-III	14.0 (8.0-20.0)	14.0 (10.0-19.3)	0.810
MDS-UPDRS	23.0 (12.0-33.0)	26.0 (17.0-32.0)	0.672
COMPASS-31 Total	17.8 (0.0-24.1)	6.8 (1.4-17.8)	0.094
BDI-II	5.0 (3.0-9.0)	4.0 (2.0-8.0)	0.676
MMSE	30.0 (29.0-30.0)	29.0 (29.0-30.0)	0.052
MoCA	28.0 (25.0-29.0)	28.0 (25.0-29.0)	0.463
Olfactory test	7.0 (4.0-9.0)	7.0 (4.0-9.5)	0.143
RBD questionnaire	3.0 (1.0-5.0)	3.0 (1.0-5.0)	0.125
LEDD (mg)	375.0 (180.0-688.0)	375.0 (258-755)	0.421
IENFD total (N°fibers/mm)	12.5 (9.8-15.5)	8.1 (4.9-10.2)	**0.002**
IENFD cervical (N°fibers/mm)	16.8 (11.5-23.4)	13.6 (6.1-16.8)	**0.032**
IENFD ankle (N°fibers/mm)	6.9 (4.3-10.2)	4.7 (2.5-5.6)	**0.002**

Abbreviations: H&Y (Hoehn and Yahr scale), MDS-UPDRS (Movement Disorder Society-Unified Parkinson's Disease Rating Scale) I, II, III and total, composite autonomic symptom scale 31 (COMPASS-31); BDI-II (Beck Depression Inventory II), MMSE (Mini-Mental State Examination), MoCA (Montreal Cognitive Assessment), RBD (Rem Behaviour Disorder), LEDD (LevoDopa equivalent Dose), IENFD (intraepidermal nerve fiber density). P-values < 0.05 were considered significant and shown in bold.
