# Supplementary material for: Alpha-synuclein oligomers and small nerve fiber pathology in skin are potential biomarkers of Parkinson’s disease
Source: NPJ Parkinsons Dis. 2021 Dec 20;7:119. doi: 10.1038/s41531-021-00262-y (PMC8688481; doi:10.1038/s41531-021-00262-y)
Supplement: Supplementary file 2 — Reporting Summary [file 41531_2021_262_MOESM2_ESM.pdf]

nature portfolio

Corresponding author(s): Giorgia Melli

Last updated by author(s): 2021.10.05

Reporting Summary

Nature Portfolio wishes to improve the reproducibility of the work that we publish. This form provides structure for consistency and transparency in reporting. For further information on Nature Portfolio policies, see our Editorial Policies and the Editorial Policy Checklist.

Statistics

For all statistical analyses, confirm that the following items are present in the figure legend, table legend, main text, or Methods section.

n/a	Confirmed	
☐	☒	The exact sample size (n) for each experimental group/condition, given as a discrete number and unit of measurement
☐	☒	A statement on whether measurements were taken from distinct samples or whether the same sample was measured repeatedly
☐	☒	The statistical test(s) used AND whether they are one- or two-sided<br>*Only common tests should be described solely by name; describe more complex techniques in the Methods section.*
☐	☒	A description of all covariates tested
☐	☒	A description of any assumptions or corrections, such as tests of normality and adjustment for multiple comparisons
☐	☒	A full description of the statistical parameters including central tendency (e.g. means) or other basic estimates (e.g. regression coefficient) AND variation (e.g. standard deviation) or associated estimates of uncertainty (e.g. confidence intervals)
☐	☒	For null hypothesis testing, the test statistic (e.g. F, t, r) with confidence intervals, effect sizes, degrees of freedom and P value noted<br>*Give P values as exact values whenever suitable.*
☒	☐	For Bayesian analysis, information on the choice of priors and Markov chain Monte Carlo settings
☒	☐	For hierarchical and complex designs, identification of the appropriate level for tests and full reporting of outcomes
☒	☐	Estimates of effect sizes (e.g. Cohen's d, Pearson's r), indicating how they were calculated

Our web collection on statistics for biologists contains articles on many of the points above.

Software and code

Policy information about availability of computer code

Data collection	SecuTrial
Data analysis	PASW Statistics 20 - SPSS

For manuscripts utilizing custom algorithms or software that are central to the research but not yet described in published literature, software must be made available to editors and reviewers. We strongly encourage code deposition in a community repository (e.g. GitHub). See the Nature Portfolio guidelines for submitting code & software for further information.

Data

Policy information about availability of data

All manuscripts must include a data availability statement. This statement should provide the following information, where applicable:

- Accession codes, unique identifiers, or web links for publicly available datasets
- A description of any restrictions on data availability
- For clinical datasets or third party data, please ensure that the statement adheres to our policy

The raw data that support the findings of this article are available on request to the corresponding author

Field-specific reporting

Please select the one below that is the best fit for your research. If you are not sure, read the appropriate sections before making your selection.

☒ Life sciences ☐ Behavioural & social sciences ☐ Ecological, evolutionary & environmental sciences

For a reference copy of the document with all sections, see nature.com/documents/nr-reporting-summary-flat.pdf

Life sciences study design

All studies must disclose on these points even when the disclosure is negative.

Sample size	Sample size was determined based on the availability of the clinical database. We estimated that a sample size of n=53 provides 80% statistical power to detect a difference of at least medium effect (Cohen's w=0.3) in the proportion of PD vs non-PD patients with abnormal alpha-syns, considering alpha=0.05 and (2-1)*(2-1) degrees of freedom.
Data exclusions	Subjects were excluded based on the exclusion criteria which were established before starting the study
Replication	Two independent investigators blinded to the diagnosis rated the measures that were reproducible, as stated in methods, page 17
Randomization	Study subjects were not randomized, since it is a case-control study, but statistical analysis took into account co-variables,as stated in statistical analysis (page 17-18)
Blinding	Investigators were blinded to the clinical conditions

Reporting for specific materials, systems and methods

We require information from authors about some types of materials, experimental systems and methods used in many studies. Here, indicate whether each material, system or method listed is relevant to your study. If you are not sure if a list item applies to your research, read the appropriate section before selecting a response.

Materials & experimental systems

n/a	Involved in the study	
☐	☒	Antibodies
☒	☐	Eukaryotic cell lines
☒	☐	Palaeontology and archaeology
☒	☐	Animals and other organisms
☐	☒	Human research participants
☐	☒	Clinical data
☒	☐	Dual use research of concern

Methods

n/a	Involved in the study	
☒	☐	ChIP-seq
☒	☐	Flow cytometry
☒	☐	MRI-based neuroimaging

Antibodies

Antibodies used	PGP9.5, Rabbit polyclonal, Abcam 15503<br>αSyn, Mouse monoclonal, Abcam 80627<br>P-αSyn at Serine 129, mouse monoclonal, Wako 015-25-191<br>5G4, mouse monoclonal, Analytik Jena Life Science 847-0102004001<br>AlexaFluor488, secondary antibody GxRb, ThermoFisher Scientific 111-545-003<br>AlexaFluor594, secondary antibody GxMs, ThermoFisher Scientific 115-585-146
Validation	We tested positive and negative samples<br>We performed staining without the primary and secondary antibodies<br>We tested different dilution of the antibodies

Human research participants

Policy information about studies involving human research participants

Population characteristics	Adult, male and female subjects with a diagnosis of Parkinson's disease or atypical parkinsonisms were enrolled as described in Methods page 13 of the manuscript
Recruitment	Patients were prospectively enrolled from the movement disorder out-patient clinic at the Neurology department (NSI-Lugano) healthy controls age-matched were recruited between patients' partners.

Ethics oversight	Cantonal ethical Committee

Note that full information on the approval of the study protocol must also be provided in the manuscript.

Clinical data

Policy information about clinical studies

All manuscripts should comply with the ICMJE guidelines for publication of clinical research and a completed CONSORT checklist must be included with all submissions.

Clinical trial registration	N/A, it is not a clinical trial
Study protocol	The study protocol is available on demand to the corresponding author
Data collection	Clinical data and biological samples were recruited in the Hospital setting, skin biopsies were analyzed in the Traslational Laboratory of the Hospital
Outcomes	Outcomes were defined based on preliminary studies and scientific pubblications and were assessed according to published and standardized protocols
